# Sex differences in functional brain networks involved in interoception: An fMRI study

**DOI:** 10.3389/fnins.2023.1130025

**Published:** 2023-03-14

**Authors:** Vincenzo Alfano, Carlo Cavaliere, Angelica Di Cecca, Giuseppina Ciccarelli, Marco Salvatore, Marco Aiello, Giovanni Federico

**Affiliations:** IRCCS SYNLAB SDN, Naples, Italy

**Keywords:** interoception, fMRI, SAQ, functional connectivity, sex differences

## Abstract

Interoception can be described as the ability to perceive inner body sensations and it is different between biological sex. However, no previous research correlated this ability with brain functional connectivity (FC) between males and females. In this study, we used resting-state functional magnetic resonance imaging to investigate FC of networks involved in interoception among males and females in a sample of healthy volunteers matched for age. In total, 67 participants (34 females, mean age 44.2; 33 males, mean age 37.2) underwent a functional MRI session and completed the Self-Awareness Questionnaire (SAQ) that tests the interoceptive awareness. To assess the effect of sex on scores obtained on the SAQ we performed a multivariate analysis of variance. A whole-brain seed-to-seed FC analysis was conducted to investigate the correlation between SAQ score and FC, and then to test differences in FC between males and females with SAQ score as a covariate. MANOVA revealed a significant difference in SAQ scores between males and females with higher values for the second ones. Also, significant correlations among interoception scores and FC in Salience network and fronto-temporo-parietal brain areas have been detected, with a sharp prevalence for the female. These results support the idea of a female advantage in the attention toward interoceptive sensations, suggesting common inter-network areas that concur to create the sense of self.

## 1. Introduction

Interoception is a multi-faced process that reflects the capability in perceiving inner body signals (e.g., Craig, 2002). It is also an umbrella term for the phenomenological experience of the body state, an experience which is ultimately a product of the central nervous system, regardless of what information the brain uses and does not use to construct this experience (Ceunen et al., 2013). Interoceptive sensibility, namely, the self-evaluation of subjective interoceptive information (Garfinkel et al., 2015), is different and less distinct than exteroceptive somatosensory stimulus (e.g., self-perceiving touch, pain or temperature; Craig, 2008). Interestingly, paying attention to one’s own bodily states is different between females and males and has been demonstrated in several independent samples (Longarzo et al., 2021). Specifically, women show higher attention to visceral states but reduced interoceptive accuracy, and this may affect how they report their own body-related sensations (e.g., Garfinkel et al., 2015; Grabauskaitė et al., 2017). These biological sex differences might be due to hormonal and physical changes experienced by women during lifespan, which reflects in the experiences of menstruation, pregnancy and menopause (e.g., Murphy et al., 2019). Visceral sensations are part of the interoceptive awareness, which is generally assessed by using the Self Awareness Questionnaire (SAQ; Longarzo et al., 2015).

Most recent research highlighted associations between brain structures and biological sex, which might be linked to different degree of interoceptive awareness. In particular, neuroimaging studies found differences in brain cytoarchitecture between females and males. For instance, Goldstein et al. (2001) reported that male brains are ten percent larger than female brains, even after intracranial volume adjustment (Cosgrove et al., 2007), this result is confirmed in a study of brain volume over the entire lifespan (Coupé et al., 2017). Also, cytoarchitectonic differences according to sex are present in brain regions such as the amygdala, hippocampus and insula (e.g., Ruigrok et al., 2014). Frontal and medial paralimbic brain areas are larger in women, while the hypothalamus, amygdala and angular gyrus appear to be larger in men (e.g., Rezzani et al., 2019). In the female population, the volume of both corpus callosum and language-related temporo-parietal regions is comparatively larger than males, whereas males have larger parietal cortical areas (Grabowska, 2017), which are linked to the visuospatial and sensorimotor function (Federico et al., 2022).

Differences between brain functional connectivity and biological sex have also been reported in literature. Resting state fMRI studies, which use the temporal correlation between fluctuations in different brain regions as a measure of intrinsic functional connectivity (FC), report stronger FC in the Default Mode Network (DMN) for females within wide brain networks, which include the posterior cingulate cortex, the precuneus and the medial prefrontal cortex bilaterally (e.g., Bluhm et al., 2008). Stronger intra-network FC in females and stronger inter-network FC in males are reported (Allen et al., 2011; Zhang et al., 2018). Also, a mixture FC differences between females and males has been reported in lobar regions (Filippi et al., 2013). Crucially, similar results were found by using Diffusion Tensor Imaging in a pediatric sample (Ingalhalikar et al., 2014), but over the healthy adults structural connectivity didn’t seem to correlate with FC (Tsang et al., 2017). Finally, FC differences among sex has been reported, with males which exhibit a greater right-brain-lateralized short-range FC as compared to females (Tomasi and Volkow, 2012).

An interesting research question is whether the above-mentioned sex-related structural/functional differences in the brain may reverberate in terms of interoceptive awareness. For instance, neuroimaging studies linked the insula and cingulate cortices to interoceptive awareness. Specifically, the anterior insular cortex has been considered as an hub which encodes and represents interoceptive information, and for this reason it has been also defined as an interoceptive cortex (Critchley et al., 2004; Craig, 2009). Notably, by adopting a microstructural point of view, the insula has reciprocal neural link with the anterior cingulate cortex (ACC), which is related to physiological information and provides autonomic responses (Craig, 2009). These regions are functionally linked within the salience network (SN), namely, a brain network critically involved in integrating highly processed sensory information with visceral, autonomic, and hedonic markers, to guide the own behavior. Such a network involves the bilateral anterior insular cortices and dorsal ACC, as well as the prefrontal cortex (PFC), supramarginal gyrus (SMG), striatum/basal ganglia, thalamus, and cerebellum (Seeley et al., 2007; Guo et al., 2012). All these sensory-related processes exhibit a decrease with aging. Indeed, numerous studies demonstrated age-related decreases in primary somatosensory cortex (Good et al., 2001; Sowell et al., 2003), insular cortex (Good et al., 2001; Resnick et al., 2003), and SN connectivity (Onoda et al., 2012).

Taking together all the above-mentioned evidence, the general picture which emerges is that very little is known about how sex-related differences in the brain may generate different levels of interoceptive awareness. Studying such differences may allow researchers to expand knowledge about how our brain perceives inner-body signals and to target specific populations in order to understand what makes them more susceptible. Therefore, the present study aims at exploring sex differences in functional brain networks involved in interoception in a large sample of healthy participants.

## 2. Materials and methods

The sample of our study includes 67 healthy subjects (34 females, mean age 44.2 ± 14.5; 33 males, mean age 37.2 ± 12.4) enrolled at the IRCCS SYNLAB SDN, and performing both an extensive neuropsychological assessment and magnetic resonance imaging (MRI). All the subject that participated in this research protocol were recruited if they met the following criteria: (i) lack of actual or past history of alcohol or drug abuse, (ii) lack of current or past history of major psychiatric illnesses, (iii) lack of history of brain injury, stroke, or any other major clinical condition, (iv) lack of current or past use of psychoactive medications. The eligibility criteria to define the participants “healthy subjects” were assessed through a brief clinical interview performed by an expert neuropsychologist. Each subject provided written informed consent that was previously approved by the local Ethics Committee of IRCCS Pascale and performed according to the ethical standards laid down in the 1964 Helsinki Declaration and its later amendments. All individuals were naïve to the scope of the study and gave their written informed consent to participate without any reward.

### 2.1. Neuropsychological assessment

All participants in the study completed the Self-Awareness Questionnaire (SAQ), a self-report tool devised to evaluate the perception of a wide range of bodily sensations and, in particular, investigate the frequency of perceiving signals from volunteer’s own body (Longarzo et al., 2015). The SAQ is composed by 28 items to be rated on a 5-point Likert scale (0 = never; 1 = sometimes; 2 = often; 3 = very often; 4 = always). The total score ranges 0–112 with higher scores represent higher interoceptive awareness. The SAQ has a bi-factorial structure; the first factor (F1) included items related to visceral sensations, whereas the second one (F2) is related to somatosensory sensations (Longarzo et al., 2015). In our research, we performed a multivariate analysis of variance (MANOVA) in order to assess the effect of biological sex, demographics parameters and SAQ scores, with a Wilks’ lambda (λ) statistic. SPSS (IBM Corp., Released 2016. IBM SPSS Statistics, Version 24.0) were used to perform statistical analyses.

### 2.2. MRI functional connectivity

A Biograph mMR 3T scanner (Siemens Healthcare, Erlangen, Germany) with a 12-channel head coil were used to acquire MR images. The MR acquisition protocol included structural and functional sequences: (1) 3D T1-Magnetization Prepared Rapid Acquisition Gradient Echo (MPRAGE), voxel size 0.8 × 0.8 × 0.8 mm^3^, Field of View (FOV) 214 × 214 mm, TR/TE/TI = 2,400/2.25/1,000 ms, scan time 5:03; and (2) Resting-state fMRI, Echo Planar Imaging-Gradient Echo sequence (EPI-GRE), voxel-size 4 × 4 × 4 mm^3^, TR/TE = 1,000/21.4 ms, 350 measurements, bandwidth: 2,230 Hz, multiband factor: 2 (Auerbach et al., 2013), scan time 6:02. fMRI data were analyzed with Functional Connectivity Toolbox (CONN v. 20b; Whitfield-Gabrieli and Nieto-Castanon, 2012) and Statistical Parametric Mapping (SPM v. 12). Both CONN and SPM were executed on MATLAB (v. 2021b). Pre-processing of fMRI data were performed with CONN using a pipeline that includes realignment, slice-timing, functional-image normalization in the Montreal Neurological Institute (MNI) space, outlier detection with ART-based scrubbing smoothing, and physiological denoising (Alfano et al., 2021). A statistical data analysis was devised to assess differences in functional connectivity (FC) between male and female participants after controlling for age, SAQ, F1 and F2 covariates. We evaluated FC differences between these two sub-groups by performing CONN-based seed-to-seed analyses, which were conducted by adopting cortical and subcortical ROIs (FSL Harvard-Oxford maximum likelihood cortical and subcortical atlas, dividing bilateral areas into left/right hemisphere for a total of 106 ROIs). Then, a regression-based inter-network FC analysis was conducted to investigate correlations between brain networks FC and SAQ, F1 and F2 scores (networks from CONN’s ICA analyses of HCP dataset for a total of eight networks with 32 subnetwork ROIs; Bar-Joseph et al., 2001). An alpha level of 0.05 was used with false discovery rate (FDR) correction for multiple comparisons (Benjamini and Hochberg, 1995) for both the seed-to-seed and network-to-network comparisons.

## 3. Results

**FIGURE 1:**
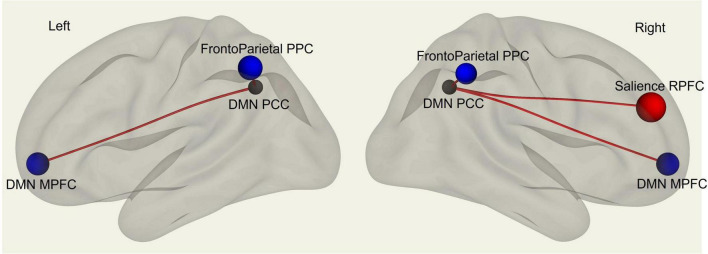
fMRI seed-to-seed three dimensional representation showing FC differences between males and females’ subjects with SAQ and age as covariates.

**FIGURE 2:**
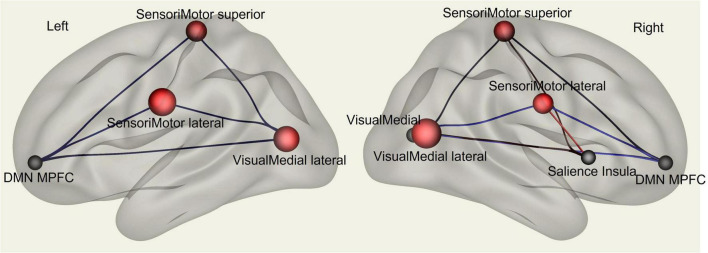
fMRI seed-to-seed three dimensional representation showing FC analysis with the SAQ considered as a continuous regressor and age as covariate.

Multivariate analysis of variance (MANOVA) showed significant differences between males and females on SAQ total score (λ = 0.03, F(hypothesis-DOF 64, error-DOF 64) = 4.5; *p* = 0.005), and on both factors, F1 (λ = 0.05, F(hypothesis-DOF 44, error-DOF 84) = 5.8; *p* = 0.002) related to visceral sensations and F2 (λ = 0.08, F(hypothesis-DOF 40, error-DOF 88) = 4.1; *p* = 0.036) related to somatosensory sensations, with females showing higher values of SAQ, F1 and F2 than males. Mean age did not differ between the two groups. By using SAQ and age as covariates, results of the whole-brain seed-to-seed network analysis showed significant differences between male and female participants (Figure 1 and Table 1). The following pattern of seed-to-seed network FC hypoconnectivity (males with a lower FC than females) was found: DMN posterior cingulate cortex (PCC) - left and right Frontoparietal network PPC (T(64) = −3.4; p-FDR = 0.018 and T(64) = −2.8; p-FDR = 0.05, respectively), DMN MPFC (T(64) = −3; p-FDR = 0.042). The following pattern of seed-to-seed hyperconnectivity (males with a higher FC than females) was found: DMN PCC - right Salience RPFC (T(64) = 4; p-FDR = 0.005). Following, the SAQ was considered as a continuous regressor and age as covariate (Figure 2 and Table 2). A moderate positive correlation among the SAQ score and the following network seeds were found: DMN (peak seed: MPFC) and the superior SensoriMotor network (T(64) = 3.3; *r* = 0.38 p-FDR = 0.045); Salience network (peak seed: right insula) and the left lateral SensoriMotor network (T(64) = −3.2; *r* = −0.37; p-FDR = 0.05); visual medial network and left and right visual lateral network (T(64) = 3.3; *r* = ; p-FDR = 0.025 and T(64) = 3.6; *r* = 0.39; p-FDR = 0.018, respectively), left and right sensorimotor lateral network (T(64) = 2.9; *r* = 0.34; p-FDR = 0.045 and T(64) = 2.7; *r* = 0.35; p-FDR = 0.048, respectively), visual occipital network (T(64) = 2.7; *r* = 0.34; p-FDR = 0.046). FC analyses with F1 and F2 scores, confirm and reflect the results and the brain areas obtained with SAQ score described above.

**TABLE 1:** Whole-brain seed-to-seed FC differences analysis between males and females’ subjects with SAQ and age as covariates.

Seed	Targets	T-score	p-FDR
DMN PCC	Salience RPFC right	4	0.005
	Frontoparietal PPC left	-3.4	0.018
	DMN (MPFC)	-3	0.042
	Frontoparietal PPC right	-2.8	0.050

Positive T-scores represent FC hyperconnectivity, while negative T scores represent FC hypoconnectivity. p-FDR, p-value corrected for multiple comparisons (Benjamini and Hochberg, 1995).

**TABLE 2:** Whole-brain seed-to-seed FC analysis with the SAQ considered as a continuous regressor and age as covariate.

Seed	Targets	T-score	p-unc	p-FDR
DMN (MPFC)	SensoriMotor superior	3.3	0.0015	0.045
Salience (insula right)	SensoriMotor lateral left	-3.2	0.0023	0.050
Visual medial	Visual lateral right	3.6	0.0006	0.018
	Visual lateral left	3.3	0.0018	0.025
	SensoriMotor lateral left	2.9	0.0047	0.045
	Visual occipital	2.8	0.0063	0.046
	SensoriMotor lateral right	2.7	0.0083	0.048

P-FDR, *p*-value corrected for multiple comparisons (Benjamini and Hochberg, 1995).

## 4. Discussion

In the present study we aimed to investigate the functional brain networks involved in interoceptive awareness in a sample of healthy males and females. To measure interoceptive awareness, we used the SAQ, namely, a neuropsychological test which measures subjective interoceptive sensibility (Longarzo et al., 2015). Behaviorally, our results highlight that females have significantly higher SAQ scores as compared to males. Specifically, females obtained higher scores than males in the SAQ total score and on both SAQ sub-factors, namely, visceral and somatosensory sensations. Then, we investigated differences in brain FC between the two groups (female vs. males), by including age and SAQ scores as covariates. Finally, we analyzed whether there were correlations between FC measures and interoceptive awareness (i.e., SAQ scores). FC results showed significant correlations among SAQ score, the right insular cortex (part of the SN) and left lateral sensorimotor network, including the somatosensory cortex. We found FC sex-related differences in interoception awareness in PCC, which is part of DMN, and in the RPFC, which is part of the SN, with males’ participants exhibiting higher FC.

Consistently with our results, most recent research underlined how the cingulate, together with the insula, contributes to elaborate interoceptive awareness, particularly in choosing the most appropriate response to perceive inner-body stimuli. Also, from a functional perspective, the cingulate showed sex differences in studies on emotional processes, which might be related to interoception (Mann et al., 2011). In particular, our findings seem to support the Critchley et al.’s (2001) two-processes hypothesis, which highlights how changes in self-perceiving bodily states may be seen as a function consisting of two hierarchical processes, namely, a first-order context-independent autonomic representational process, within the insular and somatosensory cortices, and a second-order context- and experience-dependent representational process, within the cingulate and ventromedial prefrontal cortices.

Kircher et al. (2000) reported that self-descriptive traits activate a network which includes the precuneus, superior parietal lobe, prefrontal cortex, and cingulate cortex. Coherently, our results showed that the prefrontal cortex and cingulate cortex have stronger FC in males. Also, Gusnard et al. (2001) attributed to the precuneus, cingulate and medial prefrontal areas the role of engaging continuous information and representation of the self when a person is awake and alert (Cavanna and Trimble, 2006). Sevinc et al. (2017) showed that the SN is implicated in the coordination of executive control and associative processes. Consistently with this evidence, our results suggest that the RPFC, the insular cortex, ACC/PCC, and orbitofrontal cortex may represent a crucial interoceptive hub for the human adults.

A positive correlation between SAQ scores and the FC of sensorimotor network and visual network bilaterally were found in our study. The sensorimotor network, also known as somatomotor or somatosensory network, is a large-scale brain network that primarily includes somatosensory (post-central gyrus) and motor (precentral gyrus) regions and extends to the supplementary motor areas (Smitha et al., 2017). Neural networks involved in interoceptive awareness may, therefore, extend beyond primary interoceptive regions, hence broadening somatosensory areas in order to include the posterior cingulate and hippocampus (Eckert et al., 2009). These brain regions, as well as the paracentral cortex, are critical for the representation of broader contextual information and sensorimotor engagement with the environment (Vogt et al., 2006; Jantzen et al., 2007). Coordinated neurocognitive processing among salience, visual and sensorimotor networks is important for maintaining interoceptive accuracy in older adults (Ueno et al., 2020). This evidence is in line with our pattern of positive correlation between SAQ scores and FC of sensorimotor and visual networks, by excluding sex differences from the analyses. A limitation of our study is the replicability since the SAQ as a neuropsychological test is not as spread in resting-state fMRI studies as other neuropsychological assessments more often used in neurocognitive imaging studies. Moreover, neuropsychological assessments tend to have small effect sizes (Owens et al., 2021).

To sum up, our results depict a complex framework, which concerns the interaction of multiple large-scale functional brain networks (i.e., the salience, somatosensory, and visual network) in generating interoceptive awareness. All these brain networks may concur to create a personal perspective of the own bodily status, with females showing a stronger attitude in self-perceiving and self-recognizing their internal body states. Conversely, males may pay less attention toward their body as an effect of their attitude to integrate more general, less fine, body states (Sun et al., 2015). Thus, according to this line of evidence, one may speculate that interoceptive awareness may involve more detailed, fine information processing, which is not globally distributed in the cortex. There are several potential applications of the study like considering different cut offs in calculating SAQ scores taking into account of sex and age (higher cut offs should be considered for women given their higher pre-disposition). Future research could consider a different scoring of questionnaires related to anxiety disorders if there was reported a prevalence of anxiety disorders in males or females (Jalnapurkar et al., 2018) with a link to interoceptive aspects (Pollatos et al., 2007). Sex differences in interoceptive awareness may, therefore, emerge as an effect of differences in FC of specific brain regions, which may exhibit different developmental trajectories. The following stage could calibrate interoception-related tools considering the sex differences and relying on their neuroimaging data. Future research may involve participants with mood disorders in order to investigate how interoception and neuroimaging are involved in the occurrence of such disorders and better characterize those neurocognitive processes.

## Data availability statement

The raw data supporting the conclusions of this article will be made available by the authors, without undue reservation.

## Ethics statement

The studies involving human participants were reviewed and approved by the IRCCS Pascale Naples Ethics Committee. The patients/participants provided their written informed consent to participate in this study.

## Author contributions

GF, CC, and VA conceived the study. VA, AD, and GC acquired the behavioral and fMRI data and conducted the study. GF and VA analyzed the data. VA wrote the manuscript’s first draft. GF, AD, GC, MA, MS, and CC revised the manuscript and provided the critical comments and theoretical contributions. All authors contributed to the article and approved the submitted version.
